# Exploring the Relationship between Student-Content Interaction, Student-Student Interaction, Self-Efficacy, and Learning Achievements in a Student-Centered Classroom

**DOI:** 10.12688/f1000research.165290.1

**Published:** 2025-07-18

**Authors:** Lu Zhang, Rawin Vongurai

**Affiliations:** 1 Faculty of Education, Yunnan Technology and Business University, Kunming, Yunnan, 650000, China; 2 Graduate School of Business and Advanced Technology Management, Assumption University, Bangkok, Bangkok, 10240, Thailand

**Keywords:** Student-content Interaction, Student-student Interaction, Self-efficacy, Learning Achievement

## Abstract

**Background:**

With the growing mismatch between traditional academic training and the demands of the modern workforce, student-centered education has emerged as a key reform in higher education. This study examines the relationships among student-content interaction, student-student interaction, self-efficacy, and learning achievement in student-centered classrooms at Chinese application-oriented universities.

**Methods:**

Data were collected from 524 undergraduate students via online questionnaires and analyzed using Confirmatory Factor Analysis (CFA) and Structural Equation Modeling (SEM).

**Results:**

The results indicate that both student-content and student-student interactions significantly enhance learning achievement, with self-efficacy playing a critical mediating role. Higher levels of self-efficacy were also directly associated with improved academic achievement, underscoring its central importance in student success.

**Conclusions:**

These findings highlight the value of fostering interactive learning environments and promoting the development of self-efficacy to improve educational outcomes. Educators are encouraged to implement instructional strategies that facilitate meaningful engagement, thereby supporting both the cognitive and motivational dimensions of learning. This study provides empirical evidence to inform the design of effective student-centered teaching practices in higher education.

## 1. Introduction

With the development of society, the traditional academic skills fostered by universities are no longer aligned with the demands of the modern world, as evidenced by the employment challenges encountered by Chinese university graduates and the growing societal demand for highly skilled individuals and college students ( Zhao et al., 2018). Simultaneously, higher education transitioned from a teacher-centered educational model to a student-centered approach. While the former emphasizes the delivery of content, the latter focuses on what students can learn and apply ( Manzoor et al., 2017). This shift has prompted educational reforms worldwide, and countries such as Finland, the United Kingdom, Germany, and Spain are increasingly embracing non-zoning, open, and flexible learning environments to foster student autonomy, self-regulated learning, collaboration, and digital literacy ( Hartikainen et al., 2021).

Meanwhile, there is an increasing number of application-oriented universities in China that aim to update the instructional content, teaching methods, and learning strategies to comprehensively enhance the standard of teaching and nurture high-quality, application-oriented talents with strong competitiveness and social adaptability to embody the concept of application within the educational framework ( Zhao et al., 2018). According to Coates (2005), students engaged in various activities may achieve high-quality learning. Good interactive relationships, such as group work or instructive feedback, are important for students’ engagement in learning success ( Martin & Bolliger, 2018) and high student-student interaction in courses revealed the most favorable perceptions of engagement and learning achievements ( Tsai et al., 2021). In addition, compared to students with higher self-efficacy, those with lower initial self-efficacy also had lower emotional engagement ( Martin & Rimm-Kaufman, 2015) and positive interactive teaching could promote learners’ self-efficacy ( Li & Yang, 2021). Autonomous motivation, controlled motivation, perceived self-efficacy, and perceived teaching quality are the key determinants of university students’ achievements ( Lai et al., 2021).

Therefore, this study aims to explore the relationships among student-content interaction, student-student interaction, self-efficacy, and learning achievements in a student-centered classroom within the Faculty of Education at five application-oriented universities in Southwest China, most of whom will become teachers in the future. By investigating the mediating role of self-efficacy in the connection between interactive teaching strategies and students’ learning achievements, this study will assist educational practitioners in designing effective teaching methods that meet the developmental needs of students and enhance their achievements.

## 2. Literature review and research framework

### 2.1 Literature review

**2.1.1 Student-content interaction**

Tuovinen (2000) points out that student-content interaction means that students engage with instructional materials and planned activities. The materials include textbooks, PowerPoint presentations, web pages, discussion forums, case studies, reports, and videos so on ( Su et al., 2005). Without it, there could be no education, because it is crucial progress through which the student can understand the subject of the study, interact with the content, gain opinions, and integrate cognitive structures ( Kennedy, 2020). In addition, strengthening student-content interaction has a significant impact on students’ achievements ( Bernard et al., 2009) and student achievement can be improved by providing customized learning materials ( Ipinnaiye & Risquez, 2024). Students with varying levels of self-efficacy showed different learning achievements, and those with high self-efficacy were more autonomous and engaged significantly more with the content than those with low self-efficacy ( Tseng et al., 2023).

**2.1.2 Student-student interaction**

According to Moore (1989), student-student interaction means that one interacts with another student or group members through peer cooperation, discussion, and group presentation to finish the teaching task, through which they could enhance their self-management level and encourage the development of their expertise. Elizondo and Gallardo (2020) suggested that student-student interaction referred to group activities, and peer feedback improved by incorporating factors such as social involvement, level of expertise, anonymity, training, and scoring the feedback. In addition, student-student interaction can have a direct and positive impact on learning outcomes, which subsequently affects academic achievement ( Cardoso, et al., 2011) and student-student interaction is correlated with self-efficacy and achievement ( Miller, 2015). Self-efficacy mediates the relationship between student satisfaction and student-student interaction ( Ahoto et al., 2022).

**2.1.3 Self-efficacy**

Self-efficacy is defined as a set of specific beliefs that influence a person’s ability to execute action plans in future situations, including efficacy and outcome expectations ( Bandura, 1977). According to Wang et al. (2022), self-efficacy and academic emotions mediate the relationship between interaction (learner-content and learner-learner) and learning engagement. University students can enhance their self-efficacy in designing active learning environments, which may lead to higher expectations and improved academic performance ( Latorre-Cosculluela et al., 2022). Additionally, the combined influence of learning motivation, self-efficacy, and blended learning has a substantial impact on students’ academic achievement ( Rafiola et al., 2020). Active teaching and academic self-efficacy are positive predictors of course grades, and academic self-efficacy positively influences course persistence and boosts students’ expectations of success ( Andres, 2020).

*H1:*
*Student- Content Interaction has a significant impact on Self-Efficacy.*

*H2:*
*Student-Student Interaction has a significant impact on Self-Efficacy.*

**2.1.4 Learning achievements**

Learning achievement refers to the skills and abilities acquired as a result of the learning process. They play a crucial role in education, particularly by providing teachers with insights into students’ progress in meeting learning objectives through their engagement in educational activities ( Liliana et al., 2020). Learning achievements encompass three key areas: skills and habits (psychomotor), knowledge and understanding (cognitive), and attitude (affective). Each of these can be addressed and developed based on the content provided in the school curriculum ( Rahayu et al., 2018). According to Agwu and Nmadu (2023), interactive teaching methods enhance students’ academic achievement and self-concept more effectively than traditional methods, and learner-centered activity-based strategies foster cooperative and collaborative learning, which supports students’ academic achievement and self-confidence. In addition, there is a significant positive relationship between academic self-efficacy and academic achievement among Chinese college students ( Luo et al., 2023), and self-efficacy has a positive influence on students’ academic achievement in Egypt and the Kingdom of Saudi Arabia ( Rafiola et al., 2020).

*H3:*
*Student-Content Interaction has a significant impact on Learning Achievement.*

*H4:*
*Student-Student Interaction has a significant impact on Learning Achievement.*

*H5:*
*Self-Efficacy has a significant impact on Learning Achievements.*

### 2.2 Research framework

**Figure 1.**
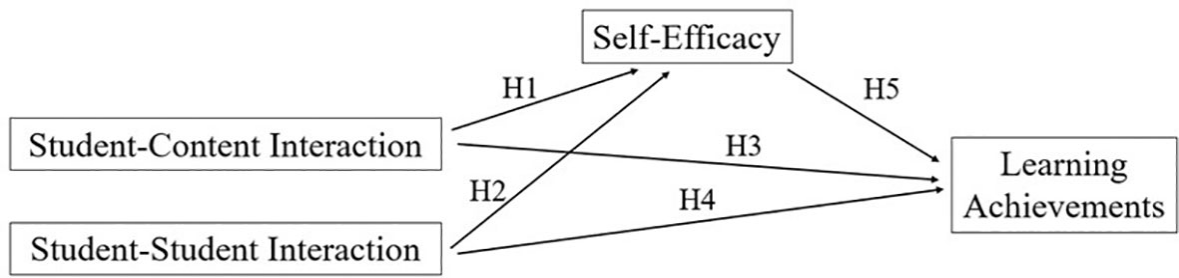
Conceptual framework.

The theoretical and conceptual frameworks outline the study’s direction, establish foundational theory, and define variables. The main objective was to enhance the relevance of the study’s findings, link them to theoretical constructs in research, and support generalization ( Adom et al., 2018). The conceptual framework shown in Figure 1 was developed by studying the theoretical framework of this research. It displays all causal relationships among variables, including student-content interaction, student-student interaction, self-efficacy, and learning achievement.

## 3. Methodology

The researcher adapted a quantitative method to conduct this study. The questionnaire was distributed using WJX, an online questionnaire tool, to students from the Faculty of Education at five application-oriented universities in China. Written informed consent was obtained from all participants via an online form embedded on the first page of the questionnaire. The consent statement clearly explained the study’s objectives, voluntary participation, anonymity, data confidentiality, and the right to withdraw at any time. Only participants who provided consent were able to proceed with the questionnaire. The questionnaire consists of three parts. The first part included screening questions to identify the respondents. The second part included demographic factors based on respondents’ gender, age, internship duration, ethnic group, and birth order. The third part consists of 5-point Likert scales to measure four different variables, ranging from extreme disagreement (1) to strong agreement (5) for the analysis of all hypotheses. In the pilot test, 30 questionnaires were distributed to assess their reliability. The researchers used Cronbach’s alpha to verify the reliability of the test. Hair et al. (2010) suggest that the alpha value should be 0.07 threshold. As a result, the questionnaire was considered reliable, as the alpha results were all above 0.07. After the reliability test, the questionnaire was distributed to 550 target respondents, and 524 responses were considered. The measurement models were designed to evaluate the validity of the variables and explore their relationships. Confirmatory Factor Analysis (CFA) was performed to assess the convergent validity within the measurement model. Finally, a Structural Equation Model (SEM) was employed to examine the overall model and analyze the effects of the variables.

### 3.1 Population and sample size

The study population consisted of university students from the Faculty of Education at five application-oriented universities in China. Most of these students are future teachers; therefore, it is meaningful to examine the relationships among interactive teaching strategies, self-efficacy, and learning achievement. On one hand, the findings can provide valuable suggestions for university instructors; on the other hand, the students themselves can apply interactive teaching strategies in their future classrooms.

These students had been studying theoretical knowledge and practical curricula for at least two semesters and had already completed internships. The researcher used the a priori sample size calculator for Structural Equation Model (SEM) from Danielsoper’s website to refer to the recommended minimum sample size ( Soper, n.d.). There were 4 latent variables and 21 observed variables, with a probability level of 0.05. The minimum recommended sample size was 342 respondents. In this study, the questionnaire was distributed among students from three different categories at five universities, with respondents evenly allocated across each category. A total of 550 participants were randomly selected, of which 524 were ultimately considered.

### 3.2 Sampling technique

In this study, the questionnaire was distributed using two methods: stratified random sampling, a probability method, and purposive sampling, a non-probability method. First, students in the Faculty of Education at five application-oriented universities fall into three categories: those from junior college (a three-year program), those who have upgraded from junior college to undergraduates (a 3+2-year program), and undergraduates (a four-year program). To select samples that allowed for the representation of the population, a proportional stratified sampling technique was applied to calculate the number of targeted respondents in each group (as shown in Table 1). This technique enables the representation of the sample for a group with a vast population ( Singh and Mangat, 1996). Purposive sampling was employed to select the respondents based on the proportionate representation of each group. The questionnaire was distributed online and included screening questions to ensure that respondents were from the Faculty of Education, had studied theoretical and practical courses for at least two semesters, and had already completed internships.

**Table 1.** Research population and sample size.

The students in the faculty of education	The target students	Research sample size
Junior college students (a three-year program)	2407	183
Students upgraded from junior college to undergraduates (a 3+2-year program)	2083	158
Undergraduates (a four-year program)	2743	209
Total	7233	550

***Note.*** Data from Student Affairs Department.

## 4. Results and discussion

### 4.1 Demographic factors

As shown in Table 2, the sample consisted of 524 valid respondents, with the data presented in terms of frequency and percentage, as detailed below:

**Table 2.** Demographic profile.

Demographic factors (N=524)		Frequency	Percent
Gender	Male	211	40.27%
	Female	313	59.73%
Age	18-19	10	1.91%
	19-20	134	25.57%
	20-21	301	57.44%
	21-22	71	13.55%
	Above 22	8	1.53%
Internship Duration	Less than 1 month	61	11.64%
	2 months	33	6.30%
	3 months	309	58.97%
	More than 4 months	121	23.09%
Ethnic group	Han	336	64.12%
	Minority Groups	188	35.88%
Birth order	1	263	50.19%
	2	159	30.34%
	3	53	10.11%
	4	49	9.35%
	Others	0	0.00%

According to Table 2, in terms of gender distribution, the majority of participants were female, comprising 59.73%, while males accounted for 40.27%. Most participants fell within the 19-21 age range, with 25.57% aged 19-20 and 57.44% aged 20-21. Smaller proportions were observed for those aged 18-19 (1.91%), 21-22 (13.55%), and above 22 (1.53%). Regarding the internship duration, the majority had completed a 3-month internship, representing 58.97% of the sample. This was followed by 23.09% who had internships lasting more than 4 months, 11.64% with internships of less than 1 month, and 6.30% with 2-month internships. The sample’s ethnic composition was primarily Han, making up 64.12%, while minority groups constituted 35.88%. In terms of birth order, participants were most commonly first-born, accounting for 50.19%, followed by second-born at 30.34%, third-born at 10.11%, and fourth-born at 9.35%.

### 4.2 Confirmatory Factor Analysis (CFA)

Confirmatory Factor Analysis (CFA), a statistical method used to validate data consistent with the conceptual models employed in this study, was initially employed to evaluate the convergent and discriminant validity of the measurement model ( Jöreskog, 1969), using factor loading, Composite Reliability (CR), and Average Variance Extracted (AVE) as the determining criteria. The guidelines suggested by Hair et al. (2006) were used to determine the significance of each item’s factor loading and acceptable thresholds for evaluating goodness of fit. The factor loadings exceeded 0.50, with p-values less than 0.05. Additionally, in accordance with Fornell and Larcker’s (1981) recommendations, the CR surpasses the 0.7 threshold, and the AVE exceeds the 0.5 benchmark, as shown in Table 3.

**Table 3.** Confirmatory factor analysis result, Composite Reliability (CR) and Average Variance Extracted (AVE).

Variables	Source of questionnaire (Measurement Indicator)	No. of item	Cronbach’s Alpha	Factors loading	CR	AVE
Student-Content Interaction (SCI)	Abou-Khalil et al. (2021)	4	0.863	0.734-0.823	0.863	0.612
Student-Student Interaction (SSI)	Abou-Khalil et al. (2021)	6	0.858	0.666-0.751	0.861	0.508
Self-Efficacy (SE)	Xiang (2020)	5	0.932	0.801-0.890	0.934	0.739
Learning Achievement (LA)	Xiang (2020)	6	0.938	0.753-0.882	0.939	0.719

***Note.*** CR = Composite Reliability, AVE = Average Variance Extracted.

The square root of the average variance extracted in Table 4 shows that all the correlation values exceed the corresponding correlations for each variable. Additionally, GFI, AGFI, CFI, NFI, and RMSEA were employed as indicators of good model fit in the CFA testing. Convergent and discriminant validity are confirmed, as the values reported in Table 5 surpass the acceptable thresholds. Consequently, this study’s convergent and discriminant validity was established. Furthermore, these measurement results provide evidence of discriminant validity and support the validity of subsequent structural model estimation.

**Table 4.** Discriminant validity.

	Factor correlations			
Variables	SCI	SSI	SE	LA
**SCI**	0.782			
**SSI**	0.652	0.713		
**SE**	0.529	0.523	0.859	
**LA**	0.592	0.578	0.750	0.848

***Note.*** The diagonally listed value is the AVE square roots of the variables.

**Table 5.** Goodness of fit.

Index	Acceptable values	Values
**CMIN/DF**	< 3.00 ( Hair et al., 2006)	2.270
**GFI**	≥ 0.90 ( Hair et al., 2006)	0.938
**AGFI**	> 0.90 ( Hooper et al., 2008)	0.914
**NFI**	≥ 0.90 ( Arbuckle, 1995)	0.958
**CFI**	≥ 0.90 ( Hair et al., 2006)	0.976
**TLI**	≥ 0.90 ( Hair et al., 2006)	0.969
**RMSEA**	< 0.05 ( Browne & Cudeck, 1993)	0.049
**RMR**	< 0.05 ( Hair et al., 2006)	0.027

***Note.*** CMIN/DF = ratio of the chi-square value to degrees of freedom, GFI = goodness-of-fit index, AGFI = adjusted goodness-of-fit index, NFI = normalized fit index, TLI = Tucker-Lewis index, CFI = comparative fit index, RMSEA = root mean square error of approximation, and RMR = root mean square residual.

### 4.3 Structural Equation Model (SEM)

Jöreskog and Sörbom (1993) described Structural Equation Modeling (SEM) as a method that employs parameters from both observed and latent variable analyses. The results for the overall model fit indices are presented in Table 5. The model fit measurement should not exceed a chi-square/degrees-of-freedom (CMIN/DF) ratio of 3, and the GFI and CFI should be higher than 0.9, as recommended by Hair et al. (2006). After running the SEMs and modifying the model using SPSS AMOS version 26, the goodness-of-fit indices were as follows: CMIN/DF = 2.270, GFI = 0.938, AGFI = 0.914, NFI = 0.958, CFI = 0.976, TLI = 0.969, RMSEA = 0.049, and RMR = 0.027 ( Table 5).

### 4.4 Research hypothesis testing results

The significance of each variable in the research model was evaluated based on the regression coefficients and R ^2^ variances. The results presented in Table 6 indicate that all hypotheses were confirmed, with significance at p = 0.05.

**Table 6.** Hypotheses testing result of the structural model.

Hypotheses	Paths	Standardized path coefficients (β)	S.E.	T-value	Tests result
H1	SE<---SCI	0.462	0.117	5.363 ***	Supported
H2	SE<---SSI	0.197	0.099	2.344 *	Supported
H3	LA<---SCI	0.139	0.056	2.183 *	Supported
H4	LA<---SSI	0.164	0.046	2.705 **	Supported
H5	LA<---SE	0.647	0.032	12.935 ***	Supported

***Note.***

* =p-value<0.05.

** = p-value<0.01.

*** = p-value<0.001.

H1: Student–content interaction has a significant impact on self-efficacy (β=0.462, SE=0.117, p<0.001). This indicates that when students actively engage with learning materials, they develop stronger beliefs about their abilities, aligning with prior research by Bernard et al. (2009) and Tseng et al. (2023).

H2: Student–student interaction has a significant impact on self-efficacy (β=0.197, SE=0.099, p<0.05). This finding supports the theories of Miller (2015) and Ahoto et al. (2022), who demonstrated that collaborative learning environments boost students’ confidence in their capabilities.

H3: Student–content interaction has a significant impact on learning achievement (β=0.139, SE=0.056, p<0.05). Engaging with content-rich materials has facilitated improved academic performance, consistent with research by Ipinnaiye and Risquez (2024).

H4: Student–student interaction has a significant impact on learning achievement (β=0.164, SE=0.046, p<0.01). This supports the findings of Cardoso et al. (2011), who highlight the critical role of peer collaboration in achieving academic success.

H5: Self-efficacy has a significant impact on learning achievements (β=0.647, SE=0.032, p<0.001), affirming Bandura’s (1977) theoretical frameworks and Luo et al.’s (2023) empirical studies. Students with higher self-efficacy demonstrate greater motivation and academic success.

### 4.5 Direct, indirect and total effects of relationships

The relationship structure among variables was analyzed using the AMOS software to assess direct, indirect, and total effects. A direct effect represents the link between the two variables, without any mediators involved in the model. An indirect effect includes at least one mediating variable that influences the relationship between two variables ( Raykov & Marcoulides, 2000). In this study, there were four variables: two independent variables, one mediator, and the dependent variable. The results of all relationships are as follows:

As Table 7 shows, student-content interaction has a significant direct positive effect on self-efficacy (β=0.462), meaning that increased interaction with content positively influences students’ self-efficacy. Student-student interaction has a weaker direct effect on students’ self-efficacy (β=0.197), indicating that interaction with peers also positively affects self-efficacy, but to a lesser extent. The R ^2^ value of 0.396 for self-efficacy shows that 39.6% of its variance is explained by student-content and student-student interactions. Self-efficacy had a strong direct positive effect on learning achievement, indicating that higher self-efficacy significantly contributed to improved learning achievement.

**Table 7.** Direct, indirect and total effects of relationships.

Variables	Self-Efficacy (SE)				Learning Achievement (LA)			
	Direct effect	Indirect effect	Total effect	R ^2^	Direct effect	Indirect effect	Total effect	R ^2^
	Standardized path coefficients (β)				Standardized path coefficients (β)			
**Student- Content Interaction (SCI)**	0.462	-	0.462	0.396	0.139	0.299	0.438	0.732
**Student-Student Interaction (SSI)**	0.197	-	0.197		0.164	0.127	0.292	
**Self-Efficacy (SE)**	-	-	-		0.647	-	0.647	

In addition, student-content interaction affects learning achievement indirectly and is mediated by self-efficacy, with an indirect effect coefficient of 0.299 and a direct effect of 0.139, showing that student-content interaction impacts learning achievement through both direct and indirect paths. Student-student interaction also impacts learning achievement indirectly via self-efficacy, with an indirect effect of 0.127 and a direct effect of 0.164, indicating both direct and mediated influences on learning. The R ^2^ value of 0.732 for learning achievement means that 73.2% of its variance is explained by student-content interaction, student-student interaction, and self-efficacy. This suggests that self-efficacy has a significant mediation effect, and that these factors collectively have a substantial influence on learning achievement.

## 5. Conclusion, recommendation and limitation

### 5.1 Conclusion

This study investigates the relationship between student-content interaction, student-student interaction, self-efficacy, and learning achievements in a student-centered classroom. Hypotheses were developed based on the conceptual framework to examine how these interactions influence students’ learning outcomes, with self-efficacy acting as a mediating factor. Confirmatory Factor Analysis (CFA) was used to assess the validity and reliability of the research model, and Structural Equation Modeling (SEM) was employed to analyze the relationships among the variables.

The findings of this study are summarized as follows. First, the student-content interaction had a significant direct impact on self-efficacy (β=0.462, p<0.001) and learning achievement (β=0.139, p<0.05). This indicates that when students actively engage with learning materials, they develop stronger beliefs about their abilities, which in turn enhances their academic performance. These findings align with prior research by Bernard et al. (2009) and Tseng et al. (2023), which emphasize the importance of meaningful content engagement in fostering academic success.

Second, student-student interaction also significantly influenced self-efficacy (β=0.197, p<0.05) and learning achievement (β=0.164, p<0.01). Collaborative learning environments such as group projects and peer discussions boost students’ confidence in their capabilities and contribute to improved academic outcomes. This supports Miller’s (2015) and Ahoto et al.’s (2022) theories, which highlight the critical role of peer collaboration in achieving academic success.

Third, self-efficacy emerged as a crucial mediator between interactive teaching strategies and learning achievement with a strong direct effect on learning outcomes (β=0.647, p<0.001). Students possessing stronger self-efficacy exhibited increased motivation and superior academic performance, supporting Bandura’s (1977) theoretical models and the empirical findings of Luo et al. (2023). The total effects analysis showed that self-efficacy explained 73.2% of the variance in learning achievement, underscoring its significant mediating role.

In conclusion, this study provides valuable insights into the mechanisms through which interactive learning strategies can enhance academic achievement. By fostering self-efficacy and promoting active engagement, educators can create more effective and inclusive learning environments that cater to students’ diverse needs. To maximize these benefits, educators are encouraged to adopt strategies, such as collaborative projects, peer reviews, personalized learning materials, and technology-enhanced tools, to stimulate meaningful interactions ( Rajaram & Rajaram, 2021). Creating a supportive learning atmosphere in which students can share ideas, receive constructive feedback, and reflect on their learning processes further strengthens their self-efficacy and academic performance ( Affuso et al., 2023). Universities should also invest in professional development programs that equip educators with interactive teaching skills to ensure their effective implementation across diverse educational settings. The validated model, supported by Confirmatory Factor Analysis (CFA) and Structural Equation Modeling (SEM), provides a robust framework for designing educational interventions that promote academic success through interactive learning practices.

### 5.2 Recommendation

First, educational institutions should implement interactive teaching strategies such as collaborative projects, problem-based learning, and peer-led discussions. These methods stimulate active participation and deeper cognitive engagement by encouraging students to share their knowledge, apply concepts, and engage in critical thinking. Research supports this approach, demonstrating that interactive learning materials significantly enhance learning achievements by fostering learner autonomy and engagement while balancing self-directed learning with structured guidance ( Tseng, Chen, & Lin, 2023). Professional development programs for educators should focus on equipping educators with the skills required to design and facilitate interactive learning experiences.

Second, as self-efficacy significantly influences academic outcomes, educators should foster supportive environments that build students’ confidence in their abilities. This can be achieved through regular constructive feedback, personalized learning goals, and the recognition of student achievements ( Luo et al., 2023; Wang et al., 2022). Creating a classroom culture in which students feel valued and empowered can enhance both motivation and academic performance, as demonstrated by recent studies on the role of teacher support in fostering self-efficacy and academic success ( Wang et al., 2022).

Third, the use of digital tools, such as learning management systems, online forums, and virtual collaboration platforms, can enhance interactions between students and course content ( Almeida & Simoes, 2023; Khan & Ahmed, 2023). Universities should invest in modern educational technologies and provide training to both students and teachers. Incorporating multimedia content, gamification, and interactive simulations can further enrich the learning experience, as demonstrated by recent studies of the positive impact of these tools on student engagement and academic performance ( Khan & Ahmed, 2023).

Finally, educational strategies should be tailored to fit learners’ cultural and contextual characteristics. Customized learning materials and culturally relevant teaching methods can help bridge cultural gaps and increase student engagement, as culturally responsive pedagogies (CRP) emphasize the importance of leveraging students’ diverse backgrounds to enhance learning outcomes ( Price et al., 2020). Conducting cross-cultural research and fostering international collaboration can further inform the development of context-sensitive educational practices, ensuring that teaching methods are both inclusive and globally informed.

### 5.3 Limitation and further study

This study has certain limitations that should be explored in future research. First, the findings are based on a specific sample size and population, which limits the generalizability of the results to other educational contexts. Future research could incorporate larger and more diverse samples across different departments and cultural settings to enhance the applicability of these conclusions. Second, the study focused on a limited set of variables, including interactive learning strategies and self-efficacy, while excluding other potentially influential factors, such as motivation, emotional intelligence, and learning environment characteristics. Therefore, future research should examine these additional variables as potential moderators of interactive learning environments. Moreover, longitudinal studies can provide deeper insights into the long-term impact of interactive teaching strategies on academic success. Collaboration among researchers, educators, and policymakers can further support the development of evidence-based best practices in higher education.

## The statement of ethical approval and consent

This study was conducted in accordance with the principles of the Declaration of Helsinki. Ethical approval was obtained from the Ethics Review Working Group of the Academic and Research Department, Yunnan Technology and Business University, China on January 4, 2024 (Reference Number: 2023042). Written informed consent was obtained from all participants via an online form embedded on the first page of the questionnaire. The consent statement clearly explained the study’s objectives, voluntary participation, anonymity, data confidentiality, and the right to withdraw at any time. Only participants who provided consent were able to proceed with the questionnaire.

## Data Availability

The data supporting this study are openly available in Figshare at http://doi.org/10.6084/m9.figshare.29319401 ( Zhang, L. & Vongurai, R., 2025). Data are available under the terms of the Creative Commons Attribution 4.0 International license (CC-BY 4.0).
