# Supplementary material for: Exploring the relationship between student-content interaction, student-student interaction, self-efficacy, and learning achievements in a student-centered classroom
Source: F1000Res. 2025 Oct 10;14:709. Originally published 2025 Jul 18. [Version 2] doi: 10.12688/f1000research.165290.2 (PMC12640484; doi:10.12688/f1000research.165290.2)
Supplement: Peer review history

# Reviewer report: Reviewer response for version 2

**DOI:** 10.5256/f1000research.189510.r402872

**Published:** 2026-10-07

**Authors:** Rani Darmayanti

**Affiliations:** 1 Mathematics Education Study Program, Universitas Nahdlatul Ulama Pasuruan, Pasuruan, East Java, Indonesia

This manuscript examines the relationships among student-content interaction, student-student interaction, self-efficacy, and learning achievement in a student-centered classroom context. The study uses Confirmatory Factor Analysis and Structural Equation Modeling to test the proposed relationships. The topic is relevant to contemporary higher education, particularly in relation to student-centered learning and the role of interaction and self-efficacy in learning outcomes.

 Overall, the manuscript has merit, and the analytical approach is generally appropriate. However, several aspects still require clarification and strengthening.

 First, the theoretical contribution should be made more explicit. The manuscript confirms several relationships that have already been widely discussed in previous studies. The authors should clarify what new theoretical or contextual contribution is provided by examining these relationships specifically in application-oriented universities.

 Second, the research gap should be articulated more clearly. The literature review provides substantial background, but the manuscript should distinguish more explicitly between the contextual, theoretical, and empirical gaps addressed by the study.

 Third, the measurement of learning achievement needs clarification. The authors should clearly explain whether learning achievement was assessed through objective academic performance, grades, self-reported perceptions, or questionnaire indicators. This distinction is important for interpreting the results appropriately.

 Fourth, more detail is needed regarding the sampling procedure. The manuscript should explain how the universities and participants were selected, the response rate, inclusion and exclusion criteria, and the extent to which the sample can be generalized to other institutional contexts.

 Fifth, the psychometric evaluation should be reported as transparently as possible. The manuscript should clearly present standardized factor loadings, composite reliability, average variance extracted, discriminant validity, and relevant model-fit indices.

 Sixth, the mediation analysis would benefit from clearer reporting. The authors should specify whether indirect effects were tested using bootstrapped confidence intervals and report these intervals where appropriate.

 Seventh, causal terminology should be used more cautiously. If the study is cross-sectional, terms such as “impact,” “influence,” or “enhances” may imply stronger causal conclusions than the design permits. More cautious language such as “is associated with” or “shows a significant relationship with” would be preferable.

 Eighth, the possibility of common-method bias should be discussed, particularly if all constructs were measured using self-report questionnaires at the same time.

 Ninth, the relatively high proportion of explained variance in learning achievement deserves further discussion. The authors should consider whether this result may partly reflect measurement characteristics or common-source effects.

 Tenth, the discussion section should do more than restate significant paths. It should explain why some relationships are stronger than others and connect these differences more directly to theory and prior research.

 Finally, the limitations section should be strengthened. The authors should explicitly acknowledge the cross-sectional design, reliance on self-report measures, institutional and geographical context, and limitations in generalizing the findings.

 The manuscript addresses an important topic and has potential value for higher education research. The concerns raised above appear addressable through revision. Therefore, I recommend **Approved with Reservations**.

Is the work clearly and accurately presented and does it cite the current literature?

Partly

If applicable, is the statistical analysis and its interpretation appropriate?

Partly

Are all the source data underlying the results available to ensure full reproducibility?

Yes

Is the study design appropriate and is the work technically sound?

Partly

Are the conclusions drawn adequately supported by the results?

Partly

Are sufficient details of methods and analysis provided to allow replication by others?

Partly

Reviewer Expertise:

Mathematics Education; Educational Technology; Student-Centered Learning; Self-Efficacy; Learning Achievement; Quantitative Research; Structural Equation Modeling (SEM)

I confirm that I have read this submission and believe that I have an appropriate level of expertise to confirm that it is of an acceptable scientific standard, however I have significant reservations, as outlined above.

# Reviewer report: Reviewer response for version 2

**DOI:** 10.5256/f1000research.189510.r422490

**Published:** 2026-01-22

**Authors:** Zhi Liu

**Affiliations:** 1 Central China Normal University, Wuhan, Hubei, China

While I appreciate the amendments made regarding the literature scope and the inclusion of the visualization figure, I feel that the depth of the theoretical discussion—particularly regarding the *mechanisms* of student-student interaction—has not yet been fully optimized to meet the previous reviewer's suggestions. To further elevate the paper's academic contribution, please consider the following points:

 Although the structural model confirms the relationships, the discussion on *why* student-student interaction yielded a weaker effect on self-efficacy compared to student-content interaction remains somewhat surface-level; could you provide a deeper sociocognitive explanation for this specific finding within your discussion section?

 The current recommendations for educators (e.g., "implement collaborative projects") are still quite generic; could you refine these into more specific, evidence-based strategies that address the unique constraints of application-oriented universities in China?

 Regarding the sampling method, while the stratified approach is described, the rationale for combining it with purposive sampling needs clearer justification to address potential concerns about selection bias and generalizability.

 To strengthen the theoretical framework regarding interaction mechanisms, I strongly recommend integrating recent studies that link social engagement to cognitive outcomes. specifically, citing "Students’ Social-Cognitive Engagement in Online Discussions: An Integrated Analysis Perspective" (Educational Technology & Society, 2023)  would provide a crucial theoretical basis for understanding how social interactions influence cognitive engagement, and "Exploring the relationships between students’ network characteristics, discussion topics and learning outcomes in a course discussion forum" (Journal of Computing in Higher Education, 2023) would offer necessary empirical support for connecting interaction patterns directly to learning achievements.

 The cultural context of Chinese application-oriented universities is mentioned, but how specifically do local educational habits (such as high-stakes testing culture or teacher-authority reliance) mediate the relationship between self-efficacy and interaction?

 In the "Limitations and Future Study" section, it would be better to explicitly mention the lack of behavioral process data (such as log data or discussion analysis) which could corroborate the self-reported survey results.

Is the work clearly and accurately presented and does it cite the current literature?

Partly

If applicable, is the statistical analysis and its interpretation appropriate?

Partly

Are all the source data underlying the results available to ensure full reproducibility?

Yes

Is the study design appropriate and is the work technically sound?

Yes

Are the conclusions drawn adequately supported by the results?

Partly

Are sufficient details of methods and analysis provided to allow replication by others?

Partly

Reviewer Expertise:

learning engagement; educational technology; online learning; blended learning; learning analytics

I confirm that I have read this submission and believe that I have an appropriate level of expertise to confirm that it is of an acceptable scientific standard, however I have significant reservations, as outlined above.

## References

[1] : Exploring the relationships between students’ network characteristics, discussion topics and learning outcomes in a course discussion forum. *Journal of Computing in Higher Education* .2023;35(3) : 10.1007/s12528-022-09335-0487-520 10.1007/s12528-022-09335-0

[2] : Students' Social-Cognitive Engagement in Online Discussions: An Integrated Analysis Perspective. *ducational Technology & Society* .2023; 10.30191/ETS.202301_26(1).0001

# Reviewer report: Reviewer response for version 2

**DOI:** 10.5256/f1000research.189510.r430043

**Published:** 2026-01-06

**Authors:** Mireya Mallén-Berdejo

**Affiliations:** 1 University of Zaragoza, Zaragoza, Spain

This study examines the relationships between student–content interaction, student–student interaction, self-efficacy, and learning achievement in student-centered classrooms within Chinese application-oriented universities. Using survey data from 524 undergraduate students, the authors apply Confirmatory Factor Analysis (CFA) and Structural Equation Modeling (SEM) to test a mediation model in which self-efficacy plays a central role. The findings indicate that both forms of interaction positively influence learning achievement, directly and indirectly through self-efficacy, which emerges as a strong mediator.

 The topic is relevant and timely, particularly in the context of student-centered reforms in higher education and teacher education. The dataset is substantial, the analytical approach is appropriate, and the article demonstrates technical competence in the use of CFA and SEM.

 Clarity of presentation and use of literature:

 The manuscript is generally well structured and clearly written, with a logical progression from introduction to conclusions. Key constructs are identifiable, and tables reporting CFA and SEM results are clearly presented. The study cites a substantial body of relevant literature, including foundational and recent empirical work.

 However, the literature review remains largely descriptive rather than analytical. While many relevant studies are cited, there is limited synthesis of contrasting findings or explicit positioning of the study within current theoretical debates on engagement and interaction. In addition, although some recent references are included, the integration of the most up-to-date literature (2024–2025), particularly on technology-enhanced and AI-supported learning environments, could be strengthened to enhance the manuscript’s contemporary relevance.

 Study design and technical soundness:

 The quantitative research design is appropriate for the stated aims. The conceptual framework is theoretically grounded, drawing primarily on interaction theory and Bandura’s self-efficacy framework. The sample size is adequate and statistically justified for SEM, and the use of CFA prior to structural modeling is methodologically sound. Overall, the study is technically robust and free from major design flaws.

 One aspect that would benefit from further clarification is the combination of stratified random sampling and purposive sampling, as this has implications for generalizability that are not fully addressed in the manuscript.

 Methods and replicability:

 The methods section provides a clear overview of the participants, instruments, data collection procedures, and analytical techniques. Reliability and validity are reported using appropriate indices (Cronbach’s alpha, CR, AVE), and model fit indices are comprehensively presented.

 That said, some methodological details remain insufficiently specified to fully ensure replication. For example, further clarification on instrument adaptation and validation within the Chinese educational context (e.g., translation procedures or cultural adaptation) would strengthen transparency. Minor inaccuracies (such as the reporting of the Cronbach’s alpha threshold) should also be corrected to avoid confusion.

 Statistical analysis and interpretation:

 The statistical analyses are appropriate and competently conducted. CFA and SEM results are clearly reported, and model fit indices fall within accepted thresholds. Hypotheses are tested systematically, and the mediation role of self-efficacy is supported by both direct and indirect effects.

 Interpretations are generally consistent with the statistical results. However, the discussion could benefit from deeper engagement with the magnitude of effects (practical significance), rather than focusing primarily on statistical significance.

 Data availability and reproducibility:

 All source data underlying the results are openly available via Figshare under a CC-BY license. This ensures transparency and allows for full reproducibility, which is a clear strength of the manuscript.

 Conclusions and support from results:

 The conclusions are broadly supported by the reported findings and accurately reflect the tested model. The emphasis on self-efficacy as a key mediating mechanism is well justified by the results.

 However, the conclusions tend to reiterate the findings rather than critically reflecting on their theoretical contribution, contextual limitations, or broader implications. A clearer distinction between empirical findings, theoretical contributions, and practical recommendations would strengthen the closing section.

Is the work clearly and accurately presented and does it cite the current literature?

Partly

If applicable, is the statistical analysis and its interpretation appropriate?

Yes

Are all the source data underlying the results available to ensure full reproducibility?

Yes

Is the study design appropriate and is the work technically sound?

Yes

Are the conclusions drawn adequately supported by the results?

Partly

Are sufficient details of methods and analysis provided to allow replication by others?

Partly

Reviewer Expertise:

Educational research; higher education; student engagement and learning processes;  quantitative research methods in education (SEM); teacher education and student-centered learning.

I confirm that I have read this submission and believe that I have an appropriate level of expertise to confirm that it is of an acceptable scientific standard, however I have significant reservations, as outlined above.

# Response

**Published:** 2026-01-06

**Authors:** lu zhang

Thank you for your supportive suggestions. I will be sure to incorporate the points you've mentioned in my future research.

# Reviewer report: Reviewer response for version 2

**DOI:** 10.5256/f1000research.189510.r430042

**Published:** 2025-11-22

**Authors:** Muhammad Kamran

**Affiliations:** 1 University of Loralai, Bolachistan, Pakistan

The paper is clear. All the Psychometrics and SEM are clearly explained. The model fit indices are in excellent ranges.  Confirmatory factor analysis result, Composite Reliability (CR) and Average Variance Extracted (AVE) are clearly explained. Discriminant validity is well documented. Goodness of fit model has been explained well. Structural Equation Model (SEM) has been portrayed well.

Is the work clearly and accurately presented and does it cite the current literature?

Yes

If applicable, is the statistical analysis and its interpretation appropriate?

Yes

Are all the source data underlying the results available to ensure full reproducibility?

Yes

Is the study design appropriate and is the work technically sound?

Yes

Are the conclusions drawn adequately supported by the results?

Yes

Are sufficient details of methods and analysis provided to allow replication by others?

Yes

Reviewer Expertise:

Psychometric Properties such EFA, CEA, SEM, Construct Validity, Discriminant Validity, and IRT.

I confirm that I have read this submission and believe that I have an appropriate level of expertise to confirm that it is of an acceptable scientific standard.

# Reviewer report: Reviewer response for version 1

**DOI:** 10.5256/f1000research.181904.r407900

**Published:** 2025-09-02

**Authors:** KAZI ENAMUL HOQUE

**Affiliations:** 1 University of Malaysia, Kuala Lumpur, Malaysia

1. Is the work clearly and accurately presented and does it cite the current literature?

 The manuscript is generally clear, well-structured, and accurately presented. Introduction-conclusion flow is reasonable. Literature review is descriptive in nature instead of analytical, and while there are many relevant references provided (2018–2023), latest research (2024–2025) is not adequately referenced.

 Recommendation: Update the literature review by synthesizing conflicting findings and incorporating more recent references, especially on digital and AI-assisted learning. Ensure consistency of reference formatting.

 2. Is the study design appropriate and does the work have academic merit?

 Assessment: Yes. The study has a good quantitative design with SEM and CFA, which is appropriate for the test of the hypothesized relationship. The conceptual framework theory basis is adequate (Bandura's model of self-efficacy, interaction theory). The sample size is sufficient and statistically sufficiently justified (N=524). My recommendation is Clarifying the rationale for combining stratified random sampling and purposive sampling because such a combination could affect generalizability.

 3. Are sufficient details of methods and analysis provided to allow replication by others?

 Generally, yes. The procedures are clearly set out (sampling process, instrument development, reliability testing, ethical clearance, SEM procedures). This should facilitate replication. Gross typographical mistakes (e.g., Cronbach's alpha criterion misspelled as 0.07 instead of 0.70) reduce readability. I suggest correcting typographical mistakes, and state whether the instruments were validated within the Chinese education environment (translation/back-translation, cultural modification).

 4. If applicable, is the statistical analysis and its interpretation appropriate?

 Yes. CFA and SEM analyses were conducted correctly with clear reporting of factor loadings, CR, AVE, and model fit indices. Statistical interpretation is satisfactory, and all hypotheses are evidence-based with p-values and coefficients. I suggest displaying mediation effects more graphically (e.g., path diagram with standardized coefficients) to facilitate interpretability. Expand the discussion to include reasons for why student-content interaction had bigger effects than student-student interaction.

 5. Are all the source data underlying the results available to ensure full reproducibility?

 Yes. The dataset has been made available on Figshare under a CC-BY license to ensure transparency and reproducibility.

  6. Are the conclusions drawn adequately supported by the results?

 Yes. The conclusions align with the findings and highlight the mediating role of self-efficacy. The conclusion part, however, in its duplication of some findings rather than identifying unique contributions is to be minimized, focus on theoretical contribution (application-oriented Chinese universities and teacher education), and differentiate practical implications from empirical findings clearly.

Is the work clearly and accurately presented and does it cite the current literature?

Yes

If applicable, is the statistical analysis and its interpretation appropriate?

Yes

Are all the source data underlying the results available to ensure full reproducibility?

Yes

Is the study design appropriate and is the work technically sound?

Yes

Are the conclusions drawn adequately supported by the results?

Yes

Are sufficient details of methods and analysis provided to allow replication by others?

Yes

Reviewer Expertise:

Education Management and Leadership

I confirm that I have read this submission and believe that I have an appropriate level of expertise to confirm that it is of an acceptable scientific standard.

# Reviewer report: Reviewer response for version 1

**DOI:** 10.5256/f1000research.181904.r405561

**Published:** 2025-09-02

**Authors:** Zhi Liu

**Affiliations:** 1 Central China Normal University, Wuhan, Hubei, China

This study provides a valuable empirical investigation into the relationships between student-content interaction, student-student interaction, self-efficacy, and learning achievement in student-centered classrooms. The research design is methodologically sound, employing CFA and SEM to validate the proposed model. However, the manuscript would benefit from deeper theoretical framing, updated literature reviews, and more nuanced discussions of contextual factors. Below are specific recommendations to enhance the rigor, relevance, and contribution of this work to the related field.

 The study can strengthen the theoretical grounding by explicitly linking student-content/student-student interactions to contemporary engagement frameworks (e.g., Community of Inquiry model) by citing recent meta-analyses. 

 The authors may explicitly position their study within contemporary social-cognitive and learning engagement theories. The current framing relies heavily on Bandura’s  self-efficacy theory but overlooks recent advancements in social-cognitive engagement frameworks (e.g., Liu et al., 2023, *Educational Technology & Society*).

 The sample is limited to Chinese application-oriented universities. The authors should address how cultural and institutional factors (e.g., high-stakes testing cultures) may mediate the observed relationships.

 The manuscript briefly mentions "technology-enhanced tools" but does not engage with recent research on AI-supported collaborative learning (e.g., Kong et al., 2025, *Internet and Higher Education*), the study may discuss how AI tools (e.g., chatbots, virtual labs) might amplify or disrupt the observed relationships, citing Kong et al.’s (2025) findings on AI’s role in scaffolding discussions.

 The recommendations for educators are generic (e.g., "use collaborative projects"). Ground them in evidence-based frameworks.

 Standardize terms (e.g., "learning achievement" vs. "academic performance") and define key constructs (e.g., "student-content interaction") using recent literature.

 The study can report full psychometric properties (factor loadings, CR, AVE) for all survey items in an appendix.

 Add a participant recruitment flowchart showing response rates and exclusions per *STROBE* guidelines. Specify exact Likert scale wording.

 Supplement p-values with Cohen's d or η² for all path coefficients to assess practical significance. Compare effect sizes to benchmarks in recent meta-analyses of educational interventions.

 Develop specific, evidence-based teaching strategies (e.g., "structured peer feedback protocols") rather than generic recommendations, such as some ground suggestions in some small teaching approaches.

 Highlight the need for temporal analysis given self-efficacy's developmental nature by discussing longitudinal studies on efficacy trajectories.

 The manuscript can add a limitations subsection acknowledging the lack of behavioral data (e.g., forum logs).

 -read for hyphen consistency (e.g., "student centered" → "student-centered").

 There are some other minor suggestions to be considered:

 Add a conceptual diagram visualizing the mediation model with standardized coefficients

 Include a table comparing findings with prior studies in similar contexts

 Discuss potential negative effects of excessive peer interaction (e.g., social loafing)

 propose specific faculty development programs for implementing the strategies

 Address potential equity issues in access to interactive learning technologies

Is the work clearly and accurately presented and does it cite the current literature?

Partly

If applicable, is the statistical analysis and its interpretation appropriate?

Partly

Are all the source data underlying the results available to ensure full reproducibility?

Yes

Is the study design appropriate and is the work technically sound?

Yes

Are the conclusions drawn adequately supported by the results?

Partly

Are sufficient details of methods and analysis provided to allow replication by others?

Partly

Reviewer Expertise:

learning engagement; educational technology; online learning; blended learning

I confirm that I have read this submission and believe that I have an appropriate level of expertise to confirm that it is of an acceptable scientific standard, however I have significant reservations, as outlined above.

# Response

**Published:** 2025-10-08

**Authors:** lu zhang

Dear editor,

 Based on the student-centered educational context in Chinese application-oriented universities, this paper investigates how classroom interaction and self-efficacy jointly influence students' learning achievement. Building upon a prior 12-week classroom intervention study that successfully used interactive activities to enhance student engagement (as confirmed by paired-sample t-tests), the current research aims to explore whether and how different forms of interaction and self-efficacy continue to impact learning achievement. The findings will subsequently inform the design of a targeted instructional intervention program to further optimize teaching and learning effectiveness in this specific educational setting.
